# The oncometabolite *R*-2-hydroxyglutarate activates NF-κB-dependent tumor-promoting stromal niche for acute myeloid leukemia cells

**DOI:** 10.1038/srep32428

**Published:** 2016-08-31

**Authors:** Jing-Yi Chen, You-Syuan Lai, Hui-Jen Tsai, Cheng-Chin Kuo, B. Linju Yen, Su-Peng Yeh, H. Sunny Sun, Wen-Chun Hung

**Affiliations:** 1 National Institute of Cancer Research, National Health Research Institutes, Tainan 704, Taiwan; 2 Division of Hematology and Oncology, Department of Internal Medicine, National Cheng Kung University Hospital, Tainan 704, Taiwan; 3 Institute of Cellular and System Medicine, National Health Research Institutes, Zhunan, Miaoli 350, Taiwan; 4 Division of Hematology and Oncology, Department of Internal Medicine, China Medical University Hospital, Taichung 404, Taiwan; 5 Institute of Basic Medical Sciences, College of Medicine, National Cheng Kung University, Tainan 704, Taiwan; 6 Institute of Molecular Medicine, College of Medicine, National Cheng Kung University, Tainan 704, Taiwan; 7 Institute of Medicine, College of Medicine, Kaohsiung Medical University, Kaohsiung 804, Taiwan

## Abstract

Mutations of isocitrate dehydrogenase 1 (*IDH1*) and *IDH2* in acute myeloid leukemia (AML) cells produce the oncometabolite *R*-2-hydroxyglutarate (*R*-2HG) to induce epigenetic alteration and block hematopoietic differentiation. However, the effect of *R*-2HG released by *IDH-*mutated AML cells on the bone marrow microenvironment is unclear. Here, we report that *R*-2HG induces IκB kinase-independent activation of NF-κB in bone marrow stromal cells. *R*-2HG acts via a reactive oxygen species/extracellular signal-regulated kinase (ERK)-dependent pathway to phosphorylate NF-κB on the Thr254 residue. This phosphorylation enhances the interaction of NF-κB and the peptidyl-prolyl *cis-trans* isomerase PIN1 and increases the protein stability and transcriptional activity of NF-κB. As a consequence, *R*-2HG enhances NF-κB-dependent expression of cytokines including IL-6, IL-8 and complement 5a to stimulate proliferation of AML cells. In addition, *R*-2HG also upregulates vascular endothelial adhesion molecule 1 and CXCR4 in stromal cells to enhance the contact between AML and stromal cells and attenuates chemotherapy-induced apoptosis. More importantly, we validated the *R*-2HG-activated gene signature in the primary bone marrow stromal cells isolated from *IDH*-mutated AML patients. Collectively, our results suggest that AML cell-derived *R*-2HG may be helpful for the establishment of a supportive bone marrow stromal niche to promote AML progression via paracrine stimulation.

Isocitrate dehydrogenease 1 (*IDH1*) and *IDH2* mutations were originally identified in acute myeloid leukemia (AML) and glioblastoma multiforme by genome-wide sequencing^1^,^2^. Subsequently, mutations of these two enzymes have also been found in other cancers including chondrosarcoma, cholangiocarcinoma, and angioimmunoblastic T-cell lymphoma^3^,^4^,^5^. IDH1 and IDH2 are key enzymes responsive for the conversion of isocitrate to α-ketoglutarate (α-KG) and mutations in the enzyme active sites gain a neomorphic function to produce high amount of *R*-2-hydroxyglutarate (*R*-2HG)^6^,^7^.

Emerging evidence suggest *R*-2HG plays an oncogenic role in promoting leukemogenesis. Delivery of *IDH1/R132H* mutant or treatment of *R*-2HG promotes cytokine-independence and blocks differentiation in hematopoietic cells^8^. In addition, inhibition of mutant IDH2 by a selective IDH2/R140Q inhibitor induces differentiation of an erythroleukemia cell line and primary human AML cells^9^. Moreover, conditional knock-in mice with *IDH1/R132H* mutation show increased numbers of hematopoietic progenitors and altered epigenetic status^10^. Retroviral infection of primary bone marrow cells with mutant IDH1 accelerates cell cycle transition and promotes leukemogenesis in mice^11^. It has also been reported that the IDH2/R140Q mutation cooperates with overexpression of *HOXA9* and *MEIS1A* or with mutations in FMS-like kinase 3 (*FLT3*) to drive acute leukemia formation in transgenic mice^12^. IDH2 mutants also cooperate with oncogenic *FLT3* or *N-RAS* to drive leukemia in mice by impairing the differentiation of cells of myeloid lineage^13^. Finally, AML patients with *IDH1* mutations have poor overall survival^14^,^15^ and AML patients with *IDH2/R172K* mutation have lower rates of complete remission and worse prognosis than those with *IDH2/R140Q* mutations^16^,^17^. The clinical impact of *IDH* mutations in AML, therefore appears to be dependent on *IDH* mutation sites and the associated mutations in other genes like *FLT3* and *NPM1*.

Under physiological conditions, the bone marrow stroma provides a supportive microenvironment for normal hematopoietic stem cells, maintaining homeostasis of self-renewal and differentiation. However, bone marrow stroma switches to a tumor-promoting role by stimulating proliferation of leukemia cells and is closely associated with the development of chemoresistance in AML patients with minimal residual disease. Reciprocal interaction between cancer and stromal cells via autocrine or paracrine stimulation by secreted factors such as growth factors and cytokines has been intensively investigated in AML. In addition, adhesion-mediated interactions through direct cell-cell contact between AML and stromal cells significantly influence therapeutic response. While the effect of *R*-2HG on the proliferation, differentiation and epigenome alteration of AML cells has been well characterized, its effect on the bone marrow stromal cells is still unclear. The response of stromal cells to *R*-2HG is expected to be different from that of AML cells because these two cell types have distinct endogenous gene expression profiles and the intracellular concentration of *R*-2HG is much lower in stromal cells. AML cells have very high intracellular level of *R*-2HG because they harbor mutant IDH1 or IDH2 which directly convert α-KG to *R*-2HG in cells. Conversely, bone marrow stromal cells seldom exhibit IDH mutations and mainly uptake extracellular *R*-2HG released by IDH-mutated AML cells. In this study, we demonstrated that *R*-2HG induced NF-κB activation in bone marrow stromal cells to create a supportive niche for AML cells.

## Results

### *R*-2HG activates NF-κB in bone marrow stromal cells

**Figure 1:**
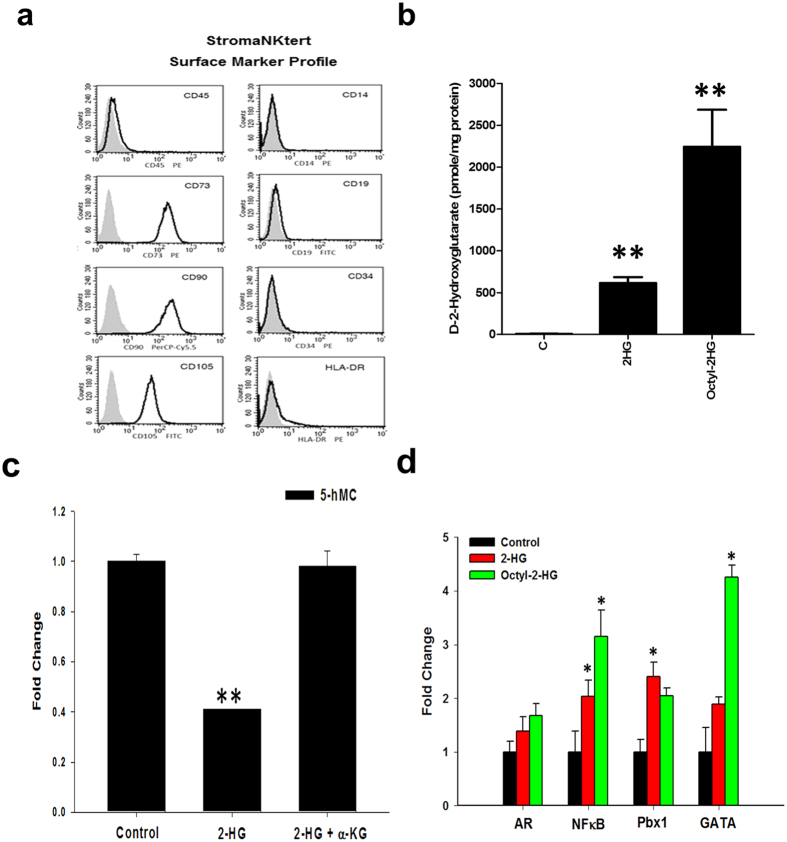
*R*-2HG activates NF-κB in bone marrow stromal cells. (**a**) Cell surface markers of StromaNKtert cells determined by flow cytometry. (**b**) StromaNKtert cells were treated with vehicle, 20 mM *R*-2HG or 1 mM octyl-*R*-2HG for 48 h and the intracellular level of *R*-2HG was quantified. (**c**) The 5-hydroxymethylcytosine level of genomic DNA was quantified using ELISA kit as described in the Methods. **p < 0.01. (**d**) StromaNKtert cells were treated with vehicle, 20 mM *R*-2HG or 1 mM octyl-*R*-2HG and the transcription factor activity was determined by array-based analysis. *p < 0.05.

We used immortalized human bone marrow StromaNKtert cells^18^ for mechanistic study and validated our results in primary bone marrow stromal cells. StromaNKtert cells express CD73, CD90 and CD105 and are negative for CD45, CD14, CD19, CD34 and HLA-DR (Fig. 1a). A number of studies used synthetic cell-permeable octyl-*R*-2HG ester to treat AML cells to mimic high intracellular concentration of *R*-2HG *in vivo*. However, this is not suitable for the study of stromal cells because stromal cells usually do not exhibit *IDH1* and *IDH2* mutations and mainly uptake *R*-2HG released from *IDH*-mutated AML cells. *R*-2HG was shown to accumulate up to ~5–35 μmol/g in *IDH*-mutated glioma tumors, which could be equivalent to 5–35 mM assuming a tissue density of 1 g/ml^19^. In addition, *R*-2HG detected by non-invasive magnetic resonance spectroscopy also confirmed this metabolite was accumulated to 5–15 mM level in tumors with *IDH* mutations^20^,^21^. The intracellular R-2HG level of stromal cells determined by mass spectrometry was very low (~8 pmol/mg protein). Treatment with 20 mM *R*-2HG or 1 mM octyl-*R*-2HG induced a 71-fold and 250-fold increase over that of control cells (Fig. 1b). The increase of intracellular *R*-2HG reduced 5-hydroxymethylcytosine level of genomic DNA by 60% and the reduction was reversed by 1 mM octyl-α-KG, indicating intracellular *R*-2HG inhibited the activity of TETs, the α-KG-dependent 5-methylcytosine hydroxylases (Fig. 1c)^22^. We addressed the transcription factor activation profile by using array-based approach and found that four transcription factors including NF-κB were consistently activated by 20 mM *R*-2HG or 1 mM octyl-*R*-2HG in StromaNKtert cells (Fig. 1d).

### *R*-2HG induces ROS/ERK-dependent phosphorylation of NF-κB to increase NF-κB stability and activity via PIN1

**Figure 2:**
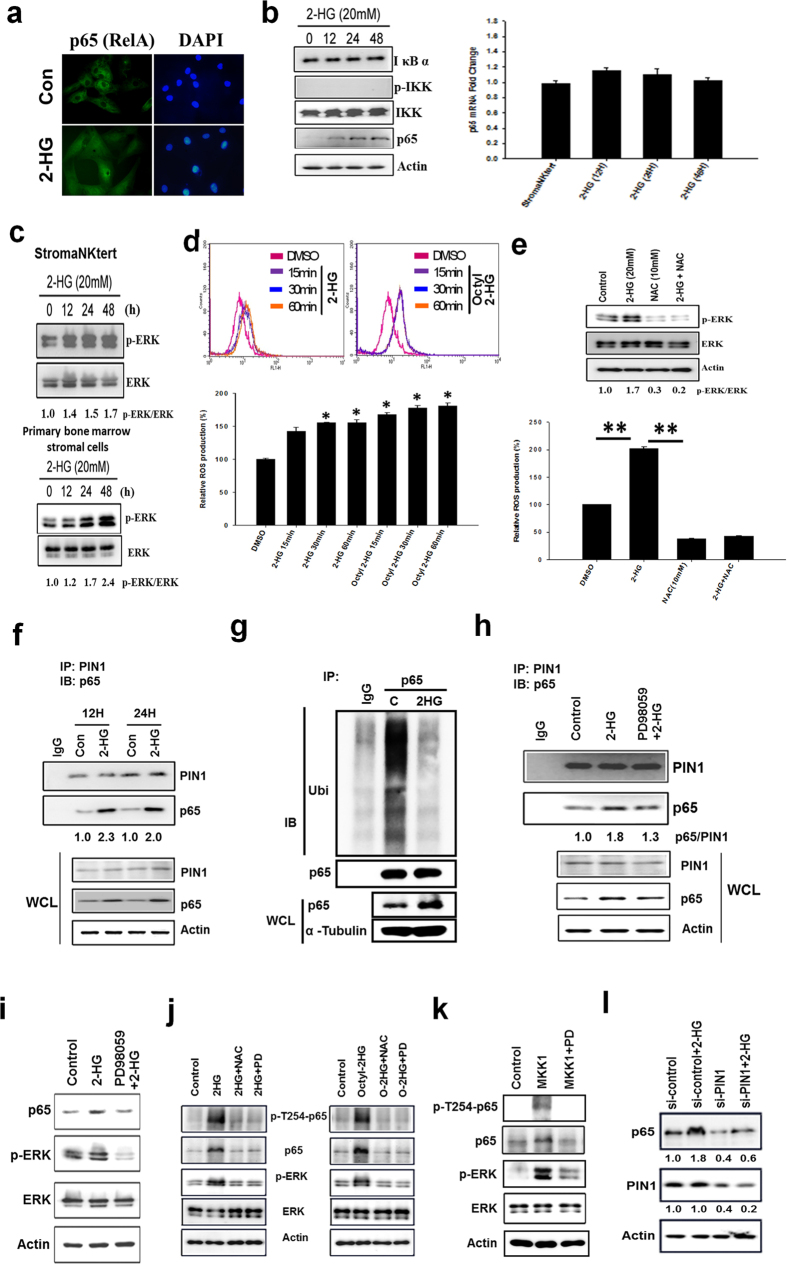
*R*-2HG promotes the interaction of PIN1 and NF-κB via ROS/ERK-dependent pathway. (**a**) Immunofluorescent staining showed the increase of nuclear entry of NF-κB in *R*-2HG-treated StromaNKtert cells. (**b**) StromaNKtert cells were treated with 20 mM *R*-2HG for different times. IκB, phospho-IKK, total IKK and p65 were investigated by western blotting. The mRNA level of p65 was also investigated by real-time RT-PCR. (**c**) StromaNKtert and primary bone marrow stromal cells (purchased from Cambrex Corporate) were stimulated with *R*-2HG for different times and ERK activation was investigated by detecting ERK phosphorylation status. (**d**) StromaNKtert cells were treated with 20 mM *R*-2HG for different times and stained by 2′,7′-Dichlorofluorescin diacetate. The intracellular level of ROS was investigated by detecting the fluorescent intensity by flow cytometry. (**e**) Cells were treated with 20 mM *R*-2HG for 24 h, followed by incubation of 10 mM N-acetylcysteine (NAC) for 6 h. ERK activation and ROS production were investigated as described in the Methods. *p < 0.05. (**f**) Cells were treated without (Con) or with 20 mM *R*-2HG for 12 h. Cellular proteins were immunoprecipitated by anti-PIN1 antibody and probed with anti-p65 antibody. WCL: whole cell lysate. (**g**) The p65 protein was immunoprecipitated and the ubiquitination status was probed by anti-ubiquitin antibody to detect the ubquitination status of p65. (**h**) Cells were pre-incubated with 10 mM PD98059 for 2 h and then treated with 20 mM *R*-2HG for another 12 h. The interaction between PIN1 and p65 was studied by immunoprecipitation/immunoblotting assay. (**i**) The p65 protein level and ERK activation were also investigated by immunoblotting. (**j**) StromaNKtert cells were pre-treated with N-acetylcysteine (NAC, 10 mM) or PD98059 (10 mM) for 2 h and then treated with 20 mM *R*-2HG for another 24 h. The phosphorylation status of Thr254 in p65 was detected by phosphor-specific antibody. (**k**) StromaNKtert cells were transfected with constitutively active MKK1 expression vector (1 μg) for 24 h and cells were treated with vehicle or PD98059 (10 mM) for another 24 h. The phosphorylation status of p65 and ERK was studied by phosphor-specific antibodies. (**l**) StromaNKtert cells were transfected with PIN1 shRNA for 24 h and treated with vehicle (control) or 20 mM *R*-2HG for another 24 h. The protein levels of PIN1 and p65 were investigated.

We focused on how *R*-2HG activates NF-κB. Immunofluorescent staining demonstrated *R*-2HG induced nuclear localization of p65 (also known as RelA) (Fig. 2a). However, *R*-2HG did not increase IκB kinase (IKK) activation and IκB degradation in StromaNKtert cells (Fig. 2b). Interestingly, p65 protein was increased in a time-dependent fashion while no change in mRNA levels was found (Fig. 2b). We found *R*-2HG activated ERK within 30 min after addition and the stimulatory effect lasted for 48 h (Fig. 2c and Supplementary Fig. S1). Similarly, *R*-2HG also activated ERK in primary bone marrow stromal cells (Fig. 2c). A recent study demonstrated that reactive oxygen species (ROS) level and NADP^+^/NADPH ratio were altered in *IDH1-R132H* conditional knock-in mice^23^. We found *R*-2HG and octyl-*R*-2HG quickly increased ROS level within 15 min after treatment and the increase lasted for at least 60 min (Fig. 2d). The ROS scavenger N-acetylcysteine suppressed basal and *R*-2HG-induced ERK activation suggesting *R*-2HG stimulates ERK activity via ROS (Fig. 2e).

We next investigated how *R*-2HG increases p65 protein stability. Because *R*-2HG did not induce IKK activation and IκB degradation, we searched for other potential signaling pathways. PIN1 has been demonstrated to bind to p65 and inhibit p65 binding to IκB upon extracellular stimulation, resulting in increased nuclear accumulation and protein stability of p65^24^. We found that the interaction between p65 and PIN1 was increased by 2-fold at 12 and 24 h after *R*-2HG addition (Fig. 2f) at which time the increase of p65 protein was detected (Fig. 2b). Immunoprecipitation assay confirmed that the binding of IκB to p65 was slightly decreased in *R*-2HG-treated stromal cells (Supplementary Fig. S2). Additionally, ubiquitination of p65 protein was reduced in *R*-2HG- or octyl-*R*-2HG-treated cells (Fig. 2g and Supplementary Fig. S3). Inhibition of ERK activity decreased the interaction of p65 and PIN1 (Fig. 2h) and the increase of p65 protein induced by *R*-2HG (Fig. 2i). The binding of p65 by PIN1 is dependent on its phosphorylation on Thr254^24^. By using bioinformatics prediction, we found the Thr254-Pro motif in p65 is a potential ERK phosphorylation site (data not shown). Indeed, phosphorylation of Thr254 was increased by *R*-2HG which could be inhibited by NAC and PD98059 (Fig. 2j). Ectopic expression of a constitutively active mitogen-activated protein kinase kinase 1 (MKK1) in stromal cells also increased Thr254 phosphorylation and PD98059 totally abolished the phosphorylation (Fig. 2k). Finally, knockdown of PIN1 reduced both basal and *R*-2HG-increased p65 protein (Fig. 2l). These data suggested that *R*-2HG induces ROS-dependent ERK activation to phosphorylate p65 and enhances PIN1-p65 interaction to increase p65 protein stability.

### Upregulation of NF-κB-dependent genes in *R*-2HG-treated bone marrow stromal cells and in *IDH*-mutated AML patients

**Figure 3:**
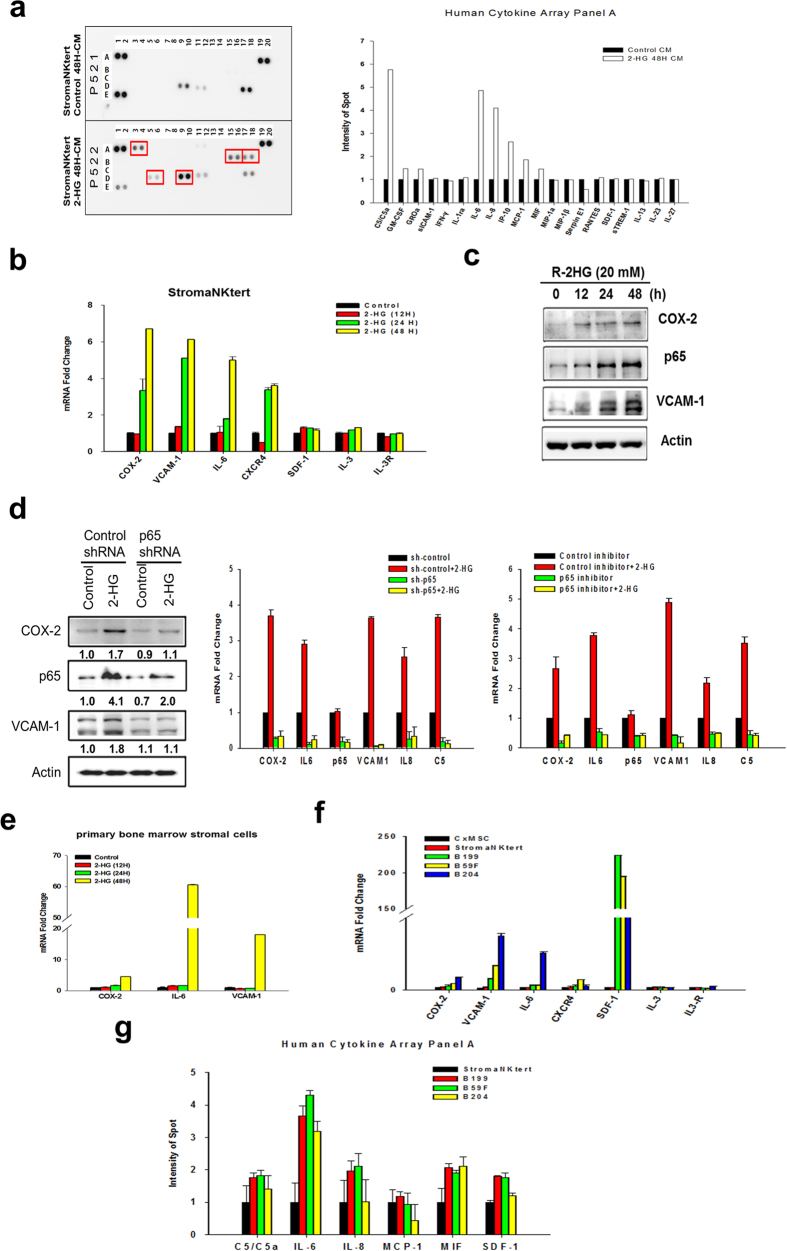
*R*-2HG stimulates NF-κB-dependent gene transcription in bone marrow stromal cells. (**a**) StromaNKtert cells were treated without (control) or with 20 mM *R*-2HG for 48 h. Conditioned medium was analyzed by cytokine array and the cytokines upregulated by *R*-2HG were indicated by red square. Averaged results of two independent experiments were shown in the right panel. (**b**) Cells were treated with 20 mM *R*-2HG for different times and the expression of NF-κB target genes were studied. *p < 0.05. (**c**) Protein level of COX-2, p65, and VCAM-1 in *R*-2HG-treated cells was investigated by Western blotting. (**d**) StromaNKtert cells were transfected with control or p65 shRNA for 24 h and were treated without or with 20 mM *R*-2HG for another 48 h. The protein level of COX-2, p65, IL-6 and VCAM-1 were studied (left panel). The mRNA level of COX-2, IL-6, VCAM-1, IL-8, and C5 was investigated by real-time RT-PCR (right upper panel). **p < 0.01 and *p < 0.05. Cells were also treated with p65 inhibitor peptide for 24 h and then stimulated with 20 mM *R*-2HG for another 48 h. The mRNA level of various NF-κB target genes was investigated by real-time RT-PCR (right lower panel). **p < 0.01 and *p < 0.05. (**e**) Primary bone marrow stromal cells (purchased from Cambrex Corporate) were treated with 20 mM *R*-2HG and the expression of COX-2, IL-6 and VCAM-1 were studied. *p < 0.05. (**f**) Expression of cytokines and cell adhesion molecules in StromaNKtert, normal primary bone marrow stromal cells and *IDH*-mutated patient’s bone marrow stromal cells (B199, B59F and B204). *p < 0.05. (**g**) Cytokine array was used to determine the cytokines released by stromal cells of three *IDH*-mutated patients. *p < 0.05.

Cytokine array showed that *R*-2HG increased complement component 5 (C5), interleukin-6 (IL-6), interleukin-8 (IL-8), interferon-γ-inducible protein 10 (IP-10) and monocyte chemotactic protein-1 (MCP-1) in the conditioned medium of StromaNKtert cells (Fig. 3a). Real-time RT-PCR validated the upregulation of C5 and IL-6 mRNA by *R*-2HG (Fig. 3b and Supplementary Fig. S4). Secreted IL-6 was also increased 5-fold by *R*-2HG (Supplementary Fig. S5). Expression of cyclooxygenase-2 (COX-2), vascular cell adhesion molecule 1 (VCAM-1) and chemokine (C-X-C motif) receptor 4 (CXCR4) which play important roles in the crosstalk between AML and stromal cells was also upregulated by *R*-2HG while interleukin 3 (IL-3) and its receptor (IL-3R) were not changed (Fig. 3b). Similar increase of COX-2 and VCAM-1 proteins in *R*-2HG- or octyl-*R*-2HG-treated StromaNKtert cells was also found (Fig. 3c and Supplementary Fig. S6). When p65 was depleted by shRNA, basal and *R*-2HG-increased mRNA expression of COX-2, IL-6, IL-8, C5 and VCAM-1 was significantly reduced (Fig. 3d). Western blot analysis confirmed the reduction of COX-2 and VCAM-1 protein in p65 knockdown cells (Fig. 3d). To further confirm this result, we used p65 inhibitor peptide which could effectively block the binding of p65 to DNA to treat cells and found that p65 inhibitor peptide also significantly suppressed basal and *R*-2HG-increased mRNA expression of COX-2, IL-6, IL-8, C5 and VCAM-1 (Fig. 3d). Treatment of *R*-2HG also upregulated the expression of COX-2, IL-6 and VCAM-1 in normal primary bone marrow stromal cells (Fig. 3e). To confirm the clinical relevance of our findings, we validated the gene signature in primary bone marrow stromal cells cultured from *IDH*-mutated AML patients. As show in Fig. 3f, COX-2, VCAM-1, SDF-1 and CXCR4 were increased in *IDH*-mutated patient’s stromal cells when compared to that of primary bone marrow stromal cells isolated from a healthy person and StromaNKtert cells. Cytokine array assay confirmed the increase of secreted C5, IL-6, and IL-8 in the conditioned medium of *IDH*-mutated patient’s stromal cells (Fig. 3g).

### *R*-2HG-induced bone marrow stromal niche enhances proliferation and chemoresistance of AML Cells

**Figure 4:**
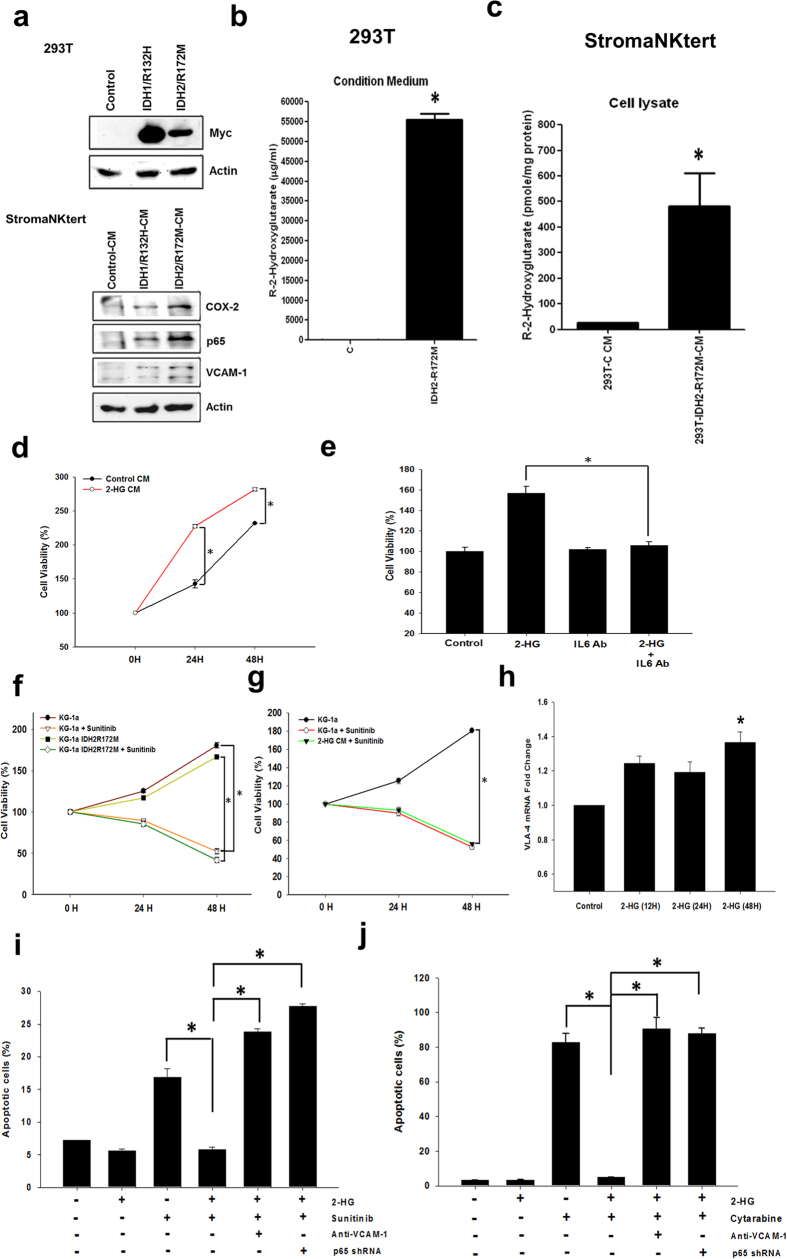
*R*-2HG-treated stromal cells enhance proliferation and chemoresistance of AML cells. (**a**) 293 T cells were transfected with different *IDH* mutants and the conditioned medium was collected to treat StromaNKtert cells. Protein level of COX-2, p65 and VCAM-1 in StromaNKtert cells was investigated. (**b**) The *R*-2HG level in the conditioned medium of control and IDH2/R172M-expressing 293 T cells was determined by mass spectrometry. *p < 0.05. (**c**) The intracellular level of *R*-2HG in stromal cells treated with the conditioned medium was also determined. *p < 0.05. (**d**) StromaNKtert cells were treated without (control) or with 20 mM *R*-2HG for 48 h. Conditioned media were collected and were was used to treat KG-1a cells. After different times, cell viability was studied by MTT assay. *p < 0.05. (**e**) StromaNKtert cells were treated without (control) or with 20 mM *R*-2HG for 48 h. Conditioned medium of *R*-2HG-treated StromaNKtert cells was pre-incubated with control IgG or IL-6 neutralizing antibody and then used to treat KG-1a cells. Cell viability was studied by MTT assay.*p < 0.05. (**f**) KG-1a cells or IDH2/R712M-expressing KG-1a cells were treated with sunitinib (1.5 μM) and cellular viability was studied. *p < 0.05. (**g**) KG-1a cells were treated without or with sunitinib (1.5 μM) in the absence or presence of conditioned medium of *R*-2HG-incubated StromaNKtert cells. *p < 0.05. (**h**) KG-1a cells were treated with 20 mM *R*-2HG and the expression of VLA-4 was studied. *p < 0.05. (**i**) KG-1a cells pre-stained with PKH26 were mixed with StromaNKtert cells. The mixed cells were treated with *R*-2HG, sunitinib or anti-VCAM-1 antibody for 48 h. In the last lane, StromaNKtert cells were first transfected with p65 shRNA for 24 h and then co-cultured with KG-1a cells in the presence of *R*-2HG and sunitinib. Apoptotic KG-1a cells (PKH26 and Annexin V double-positive cells) and viable KG-1a cells (PKH26-positive and Annexin V-negative cells) were analyzed by flow cytometry. *p < 0.05. (**j**) Chemoresistance assay was performed as described in (**i**) except the cytotoxic drug used was cytarabine.

We ectopically expressed Myc-tagged *IDH1/R132H* or *IDH2/R172M* mutants in 293 T cells or KG-1a AML cells and collected the conditioned medium to treat StromaNKtert cells. As expected, the conditioned medium increased protein level of COX-2, p65 and VCAM-1 in stromal cells (Fig. 4a and Supplementary Fig. S7). The *R*-2HG level in the conditioned medium of IDH2/R172M-expressing cells reached mM level (Fig. 4b) and the intracellular level of *R*-2HG in the medium-treated stromal cells was significantly increased by about 50-fold (Fig. 4c). Exogenous addition of *R*-2HG or ectopic expression of *IDH2/R172M* mutant did not stimulate the proliferation of KG-1a cells (Supplementary Fig. S8). Conversely, the conditioned medium of *R*-2HG-treated stromal cells was able to promote the proliferation of KG-1a cells (Fig. 4d). Because IL-6 was highly upregulated by *R*-2HG, we tested the effect of IL-6 on KG-1a cells and found that recombinant IL-6 protein stimulated the growth of KG-1a cells (Supplementary Fig. S9). Incubation of the conditioned medium with anti-IL-6 antibody significantly attenuated the growth-promoting activity indicating that *R*-2HG released by AML cells increased the production of IL-6 from stromal cells to enhance the proliferation of leukemia cells via a paracrine stimulation (Fig. 4e). The effect of *R*-2HG on chemosensitivity was also tested. A multiple kinase inhibitor sunitinib induced apoptosis of KG-1a cells (Fig. 4f). Overexpression of *IDH2/R172M* mutant in KG-1a cells could not rescue sunitinib-induced cell death indicating *IDH*-mutated AML cells are still sensitive to sunitinib (Fig. 4f). Interestingly, *R*-2HG-treated stromal conditioned medium also could not prevent the cytotoxic effect of sunitinib (Fig. 4g). We hypothesized that a direct cell-cell contact is needed to confer resistance to chemotherapy-induced apoptosis. We investigated the role of VCAM-1, a cell adhesion molecule highly up-regulated by *R*-2HG in our study. Expression of very late antigen-4 (VLA-4), the VCAM-1 ligand, was detected in KG-1a cells and was increased by *R*-2HG (Fig. 4h). We labeled KG-1a cells with the fluorescent dye PKH26 and mixed them with StromaNKtert cells. As shown in Fig. 4i, the percentage of dead KG-1a cells (Annexin-V and PKH26 double-positive cells compared to PKH26-positive cells) was increased about three-fold by sunitinib. Co-incubation of *R*-2HG reduced apoptosis to the control level. Inhibition of VCAM-1 by specific neutralizing antibody restored the cytotoxicity of sunitinib. Additionally, depletion of p65 in stromal cells restored the cytotoxicity of sunitinib indicating the protective effect is NF-κB-dependent (Fig. 4i). Similar experiments were conducted by using cytarabine, a chemotherapeutic drug for AML. As shown in Fig. 4j, cytarabine induced significant apoptosis in KG-1a cells and co-culture with stromal cells effectively attenuated the cytotoxicity of cytarabine. Inhibition of the cell contact by anti-VCAM-1 neutralizing antibody or knockdown of p65 in stromal cells restored the cytotoxic effect of cytarabine. These data suggested that upregulation of VCAM-1 by *R*-2HG in stromal cells increased the chemoresistance of AMLs by enhancing the adhesion of AML cells via a VLA-4/VCAM-1 interaction.

## Discussion

Mutations of *IDH1* and *IDH2* have great impact on the development and progression of AML and are attractive targets for cancer treatment. Recent studies have elucidated the role of *R*-2HG in regulating the proliferation, differentiation and cytokine independence of AML cells via inhibition of α-KG-dependent dioxygenases to control epigenome of cancer cells^6^. To the best of our knowledge, this study provides the first evidence showing the effect of *R*-2HG on bone marrow stromal cells. We demonstrate that AML cell-derived *R*-2HG may be helpful for the establishment of a tumor-promoting bone marrow stromal niche for AML cells by producing growth-proliferating cytokine (IL-6) and enhancing cell-cell interaction (VLA-4/VCAM-1) to increase proliferation and chemoresistance. More importantly, we identified the gene signature induced by *R*-2HG in StromaNKtert cells and validated it in primary bone marrow stromal cells isolated from *IDH*-mutated AML patients. These results suggest that *R*-2HG released from *IDH*-mutated AML cells may alter tumor microenvironment to promote AML progression. The importance of bone marrow stromal cells in the therapy of AML has been intensively investigated recently. Co-culture of JAK2^V617F^-mutated leukemia cells with bone marrow stromal cells significantly increased the resistance to a JAK2 inhibitor^25^. The protective activity of stromal cells is mediated by released cytokines via a paracrine effect. Interestingly, IL-6, an *R*-2HG-upregulated cytokine identified in our study, also plays a critical role in JAK2 inhibitor resistance. Another study showed that stromal cells diminish the cytotoxic effect of multiple kinase inhibitors that target *FLT3*-mutated AML cells and the JAK inhibitors could override stromal protection to potentiate the anti-cancer activity of FLT3 inhibitors^26^. AML cells also induce expression and secretion of growth arrest-specific 6 (GAS6), the ligand of AXL tyrosine kinase receptor, in bone marrow stromal cells and GAS6 in turn stimulates the proliferation, survival and chemoresistance of AXL-expressing AML cells^27^. A combination of AXL inhibitors and chemotherapy yields an additive therapeutic effect on AML cells. All these results suggest simultaneous targeting of AML and stromal cells may improve therapeutic efficacy. Results of this study suggest that IDH inhibitors may have a dual benefit in AML treatment by blocking the proliferation of AML cells directly and disrupting the *R*-2HG-induced bone marrow niche indirectly. Currently, two clinical trials are undergoing to investigate the combination of IDH inhibitors and chemotherapeutic drugs in AML treatment (NCT02632708 and NCT02577406, ClinicalTrials.gov) and results of these trails may provide new therapeutic strategies.

Activation of NF-κB by *R*-2HG via a PIN1-dependent pathway is another new finding in this study. We found that *R*-2HG enhances IKK-independent and ERK-dependent phosphorylation of NF-κB to promote the binding of PIN1 to increase p65 protein stability and to activate NF-κB-mediated gene transcription. Although the phosphorylation of Thr254 in p65 has been demonstrated to play a critical role in its binding to PIN1, the upstream kinases that induce phosphorylation of this residue are still unknown. Two lines of evidences led us to consider ERK as a potential candidate. First, ERK catalyzes the phosphorylation of Ser/Thr residues that occur in the sequence Ser/Thr-Pro and the Pro residue at the P + 1 position is the most reliable primary sequence determinant of ERK^28^. Bioinformatics prediction indeed suggested that the Thr254-Pro consensus sequence of p65 is a strong phosphorylation motif of ERK (data not shown). Second, ectopic expression of a constitutively active MKK1 increased ERK activation and Thr254 phosphorylation which could be totally abolished by PD98059 (Fig. 2k). We demonstrate for the first time that ERK is an upstream kinase that phosphorylates Thr254 in p65 to activate its transcriptional activity.

The involvement of additional signaling molecules in *R*-2HG-induced NF-κB activation could not be underscored. Recently, a histone demethylase KDM2A has been identified to be a negative regulator of NF-κB^29^. Because the enzymatic activities of the JmjC histone demethylases including KDM2A are potently suppressed by *R*-2HG^30^, it is possible that *R*-2HG may inhibit KDM2A and relieves its inhibition on NF-κB activity. The role of KDM2A in *R*-2HG-increased NF-κB activation in stromal cells warrants further investigation.

When this manuscript was under submission, another study published online very recently demonstrated that circulating *R*-2HG produced by IDH-mutated AML cells acts in a paracrine manner to drive the expansion of different leukemic and preleukemic clones that may express wild-type IDH1 to promote leukemogenicity^31^. The results of this study are consistent with our conclusion that extracellular *R*-2HG can stimulate bone marrow cells including preleukemic cells, stromal cells, endothelial cells etc to modulate the tumor microenvironment during AML progression. Collectively, we demonstrate a novel oncogenic function of *R*-2HG in AML by activating NF-κB-dependent gene transcription in stromal cells which may be helpful for the establishment of a tumor-promoting stromal niche for AML cells.

## Methods

### Cell culture

KG-1a cells were obtained from the Bioresource Collection and Research Center (BCRC, Taiwan) and cultured in IMDM medium containing 15% fetal bovine serum (FBS). Bone marrow StromaNKtert cells were purchased from RIKIN (ID: RCB 2350, Japan) and were cultured in IMDM medium containing 10% FBS. Human bone marrow mesenchymal stem cells were purchased from Cambrex Corporate (East Rutherford, NJ, USA) and cultured according to manufacturer’s instructions. Bone marrow samples were collected from three IDH-mutated AML patients in accordance with the regulations on human trials of Ministry of Health and Welfare, Republic of China. Preparation and culture of bone marrow mesenchymal stem cells from were performed as described previously^32^. This study was approved by the Institutional Review Board of China Medical University Hospital and signed informed consent was obtained from all patients. The patient’s information was shown in Supplementary Table 1. All experiments were performed in accordance with the guideline for Research of Human Sample of National Health Research Institutes.

### Materials

*R*-2HG was purchased from Santa Cruz Biotechnology (Santa Cruz, CA, USA). IL-6 recombinant protein was obtained from PeproTech (Princeton Business Park, NJ, USA). Sunitinib was kindly given by Dr. Ann-Lii Cheng (National Taiwan University). PKH26 were purchased from Sigma-Aldrich (St. Louis, MO, USA). Octyl-*R*-2HG and octyl-α-KG were obtained from Cayman Chemical Company (Ann Arbor, MI, USA). The NF-κB p65 inhibitor peptide was purchased from Novus Biologicals (Littleton CO, USA). The expression vectors pCMV6-Entry-IDH1/R132H and pCMV6-Entry-IDH2/R172M were kindly provided from Dr. Hai Yan (Duke University Medical Center). The target sequence of the p65 shRNA is: GCCTTAATAGTAGGGTAAGTT. The antibodies and primers used in this study were listed in Supplementary Table 2.

### Characterization of StromaNKtert cells

Differentiation studies including adipogenesis, osteogenesis and chrondrogenesis were performed as previously described to check the mesenchymal stem cell properties of StromaNKtert cells^33^,^34^.

### Determination of intracellular *R*-2HG

StromaNKtert cells were treated with vehicle, 20 mM *R*-2HG or 1 mM Octyl-*R*-2HG for 48 h and the metabolites were extracted from cells as previously described^35^. *R*-2HG was measured using UPLC coupled with a Xevo-TQ mass spectrometer. Liquid chromatography was performed on Acquity UPLC system (Waters) using a BEH C18 column (1.7 μm, 2.1 mm × 100 mm, Waters Corporation). UPLC linear gradient conditions were: 0–3 min, 1% B; 3–5 min, 30% B; 6–8.5 min; 99% B; and 9.5–12 min 99% A [solvent system A: water/formic acid (100:0.1, vol/vol); B: acetonitrile/formic acid (100:0.1, vol/vol)]. The injection volume was 2 μL, and the column temperature was maintained at 35 °C. Mass spectrometry detection was performed by using a Xevo™ Triple Quadrupole MS (Waters Corporation) equipped with an electrospray ionisation (ESI) source operating in negative ionization mode. The online MS analysis was at the Multiple Reaction Monitoring (MRM) mode. Parameters for the cone energy and collision energy for R-2HG are: parent ion 147.02 m/z, daughter ion 129.02 (m/z), cone energy 14 V, collision 12 V. Quantification of R-2HG was done using Target Lynx software (Waters Corporation).

### RNA extraction and quantitative reverse transcription-PCR analysis

Total RNA was isolated from cells using an RNA extraction kit (Geneaid, Taiwan), and 1 μg of RNA was reverse-transcripted to cDNA. Target mRNAs were quantified using real-time PCR reactions with SYBR green fluorescein and actin was served as an internal control. cDNA synthesis was performed at 95 °C for 5 min, and the conditions for PCR were 30 cycles of denaturation (95 °C/45 sec), annealing (60 °C/45 sec), extension (72 °C/45 sec), and 1 cycle of final extension (72 °C/10 min).

### Immunoprecipitation and western blot analysis

Cells were treated with or without 20 mM *R*-2HG or 1 mM Octyl-*R*-2HG ester for different times in serum-free medium. The cells were harvested with a RIPA buffer, and cellular lysate was incubated with anti-PIN1 antibody overnight at 4 °C with rotation. Immunocomplexes were pulled down by Protein-G agarose bead, washed with RIPA buffer 3 times and eluted with a sample buffer in boiled water for 10 min. The eluted samples were subjected to SDS-PAGE separation, and proteins were transferred to nitrocellulose membranes. Finally, the blots were probed with anti-p65 antibody and developed by enhanced chemiluminescence reagent. For the study of p65 ubiqutination, the cellular lysate was incubated with anti-p65 antibody overnight at 4 °C with rotation. The eluted samples were subjected to SDS-PAGE separation and the ubiqitination status of p65 protein was detected with anti-ubiqutin antibody.

### Quantification of IL-6

1 × 10^6^ stromalNKtert cells were treated 20 mM *R*-2HG in serum-free growth medium for different times. The conditioned medium of stromaNKtert cells was harvested and centrifuged to remove cells debris. The concentrations of IL-6 in the conditioned medium were detected by using Human IL6 Quantikine Immunoassay kit (R&D Systems, Inc. Minneapolis, USA) according to the manufacturer’s procedure.

### Establishment of mutant IDH stable cell lines

To establish IDH1/R132H or IDH2/R172M-expressing cell lines, KG-1a cells (1 × 10^6^) were re-suspended in buffer R containing 2 μg of IDH1/R132H or IDH2/R172M plasmids and transfected by using NeonTM microporation transfection system at room temperature under the condition of 1200 V, 20 ms, and 2 pulses. The transfected cells were cultured at 37 °C in a 5% CO2-humidified atmosphere and selected with G418 (600 μg/mL) to establish stable clones.

### Cell viability assay

KG-1a cells (1 × 10^5^/well) were plated into 96-well plates and treated with or without conditioned medium of *R*-2HG-incubated StromalNKtert cells. After different times, MTT reagent was added into the wells and cells were incubated at 37 °C for 4 h. DMSO was added to resolve the purple precipitate and the absorbance at 570 nm was measured. To study the role of IL-6 on stromal cell-induced KG-1a cell proliferation, conditioned medium of *R*-2HG-incubated StromalNKtert cells was pre-incubated with IgG or anti-IL-6 antibody (1 μg/ml) and was used to treat KG-1a cells. After 24 h, the viability of KG-1a cells was analyzed by MTT assay.

### Assay for reactive oxygen species (ROS) production

Cells were treated with 20 mM *R*-2HG for 24 h flowed by incubation of 10 mM N-acetyl-L-cysteine (NAC) for 6 h. Cells were stained with 10 μM 2′,7′-Dichlorofluorescin diacetate and the production of ROS was immediately detected by using flow cytometry.

### Immunofluorescent staining

StromaNKtert cells were treated with or without 20 mM R-2HG for 24 h and fixed with 3.7% formaldehyde for 15 min at room temperature. Cells were washed twice with PBS and permeabilized by 0.1% Triton X-100 solution for 10 min. After permeabilization, cells were incubated with 0.05% BSA solution to block nonspecific binding. Anti-p65 antibody was added and incubated at room temperature for 1 h. After extensively washing, Alexas Fluro 488 anti-rabbit IgG was added and incubated for another 1 h. Cell nuclei were stained with DAPI solution. Finally, coverslips were washed twice by PBS and placed in mounting solution. The fluorescent image was observed under a fluoresencet microscope.

### Chemosensitivity assay

For chemotherapy-induced apoptosis assay, StromalNKtert cells were seeded in 12 well plates and cultured for overnight. KG-1a cells pre-stained with PKH26 red fluorescent dye for 10 min and washed with PBS three times to remove no binding. PKH26-stained KG-1a were seeded onto StromaNKtert cells. The cells were treated with or without *R*-2HG (20 mM), sunitinib (1.5 μM) or cytarabine (3 μM) for 48 h. Apoptotic cells were stained with Annexin V-FITC Apoptosis Kit. Apoptotic KG-1a cells (PKH26 and Annexin V double-positive cells) and viable KG-1a cells (PKH26-positive and Annexin V-negative cells) were analyzed by flow cytometry.

### Determination of 5-hydroxymethylcytosine

The 5-hydroxymethylcytosine of genomic DNA was detected by using Quest 5-hmC TM DNA ELISA Kit (ZYMO Research Corp. Irvine, USA). StromaNKtert cells were collected and genomic DNA was extracted with Tissue & Cell Genomic DNA purification kit (GMbiolab Co. Ltd, Taiwan). Genomic DNA was denatured at 98 °C for 5 min and single-stranded DNA was diluted to final concentration 1 ng/μL. Diluted DNA (100 μL) was added into each well coated with anti-5-hydroxymethylcytosine polyclonal antibody and incubated at 37 °C for 1 h. After incubation, the plates were washed 3 times and incubated with horseradish peroxidase (HRP)-conjugated anti-DNA antibody at 37 °C for 30 min. Developing reagent was added and allowed for color development at room temperature for 10 min. Finally, the absorbance was measured at 450 nm with ELISA plate reader.

### Transcription factor activity array

The profile of transcription factors activated in R-2HG-treated StromaNKtert cells was screened with TF Activation Profiling Array I (Signosis, Inc., Santa Clara, USA). Nuclear extract was extracted with Nuclear Extraction Kit (Signosis, Inc., Santa Clara, USA). Five μg of nuclear extract was mixed with TF binding buffer mix (15 μL) and TF probe mix (3 μL) and incubated at room temperature for 30 min to form TF probe-DNA complex. The TF probe-DNA complex was separated by isolation column. The complex was denatured at 98 °C for 5 min and the denatured probe was hybridized with the 96-well hybridization plate at 42 °C overnight. Plates were washed three times and incubated with streptoavidin-HRP conjugate. After incubation at room temperature for 45 min, substrate solution was added to each well and incubated for 1 min. The luciferase activity was detected with a luminometer.

### Cytokine array

The conditione medium of control or *R*-2HG-treated StromaNKtert cells was collected and the particulates were removed by centrifugation. The cytokines in conditioned medium were detected with Human Cytokine Array Panel A (R&D systems, Inc., Minneapolis, USA). Membranes were blocked with 2 ml of Array Buffer 4 and incubated at room temperature for 1 h. Human Cytokine Array Panel A Detection Antibody Cocktail was added each well and incubated overnight at 4 °C on a rocking platform. After incubation, the membranes were washed and incubated with streptoavidin-HRP at room temperature for 30 min. Chemiluminescent detection reagents were incubated with membrane for 1 min and the signal intensities on the membranes were detected with BioSpectrum Imaging System (UVP LLC, Upland, USA).

### Statistical Analysis

All experiments were done in triplicate and were repeated two to three times. Results were expressed as Mean ± S.E. P values were calculated using the Student’s t test and p < 0.05 was considered as statistically significant.

## Additional Information

**How to cite this article**: Chen, J.-Y. *et al*. The oncometabolite *R*-2-hydroxyglutarate activates NF-κB-dependent tumor-promoting stromal niche for acute myeloid leukemia cells. *Sci. Rep.* **6**, 32428; doi: 10.1038/srep32428 (2016).

## Supplementary Material

Supplementary Information
