# Supplementary material for: The oncometabolite R-2-hydroxyglutarate activates NF-κB-dependent tumor-promoting stromal niche for acute myeloid leukemia cells
Source: Sci Rep. 2016 Aug 31;6:32428. doi: 10.1038/srep32428 (PMC5006242; doi:10.1038/srep32428)
Supplement: Supplementary Information [file srep32428-s1.pdf]

Manuscript title: The oncometabolite *R*-2-hydroxyglutarate activates NF-κB-dependent tumor-promoting stromal niche for acute myeloid leukemia cells

Authors: Jing-Yi Chen, You-Syuan Lai, Hui-Jen Tsai, Cheng-Chin Kuo, B. Linju Yen, Su-Peng Yeh, H. Sunny Sun, Wen-Chun Hung

Supplemental Figures and Figure Legends

Figure S1 to S9

Supplemental Tables

Table S1 to S2

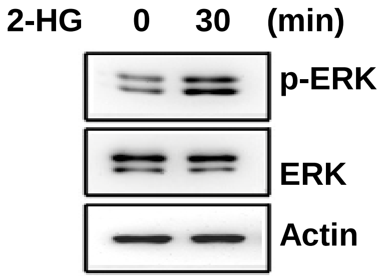

Supplementary Figure S1.

***R*-2HG induced ERK activation in stromal cells within 30 min after addition.**

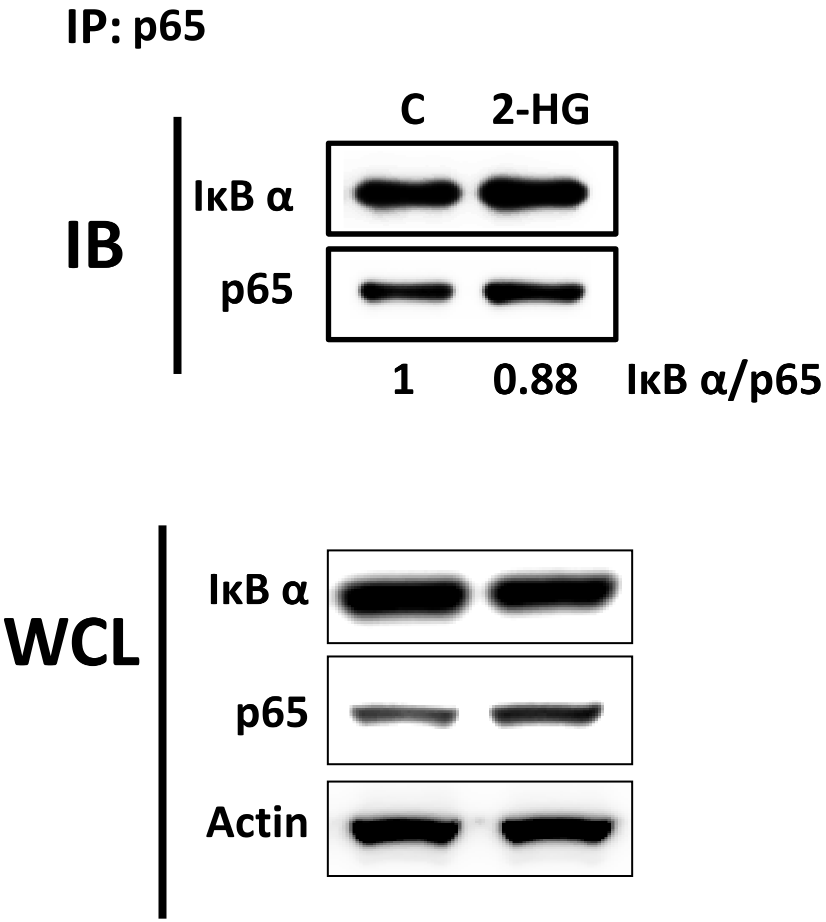

Supplementary Figure S2.

The binding of IκB to p65 was slightly decreased in *R*-2-HG-treated bone marrow stromal cells

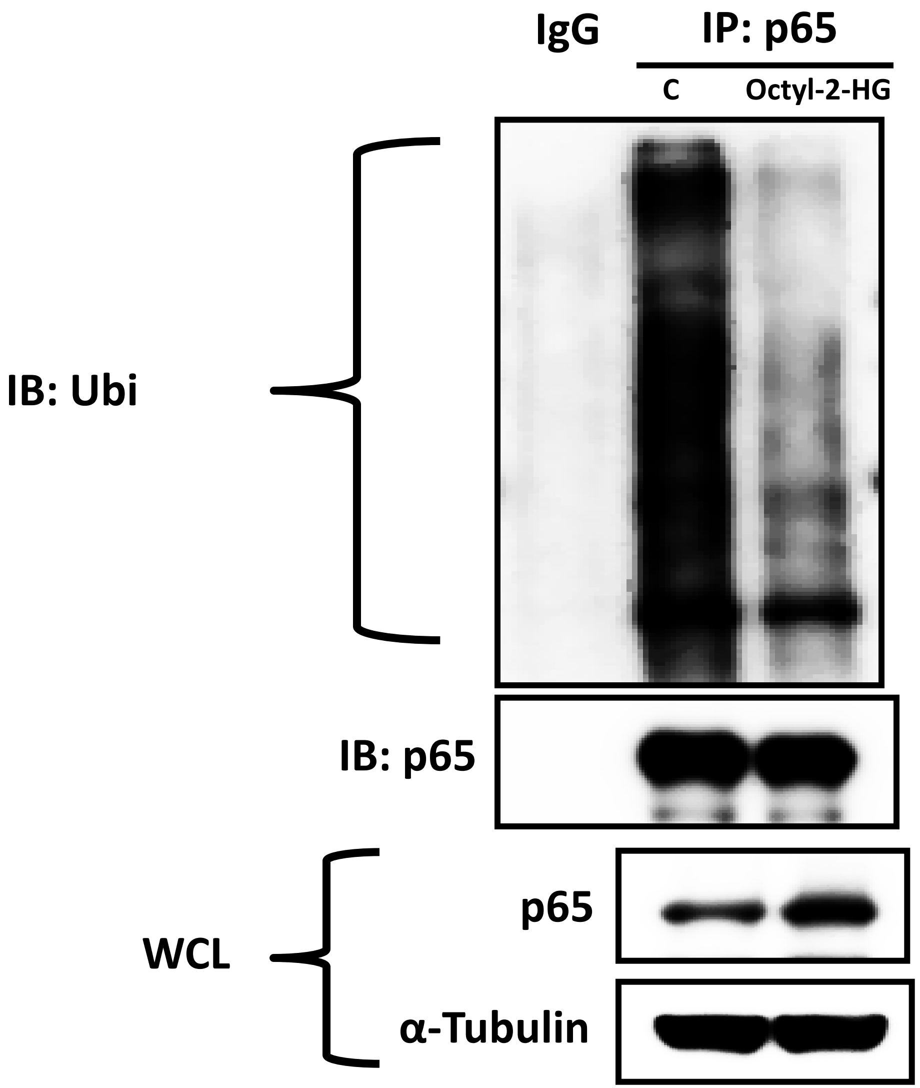

Supplementary Figure S3.

Octyl-*R*-2-HG reduced p65 ubiquitination in stromal cells.

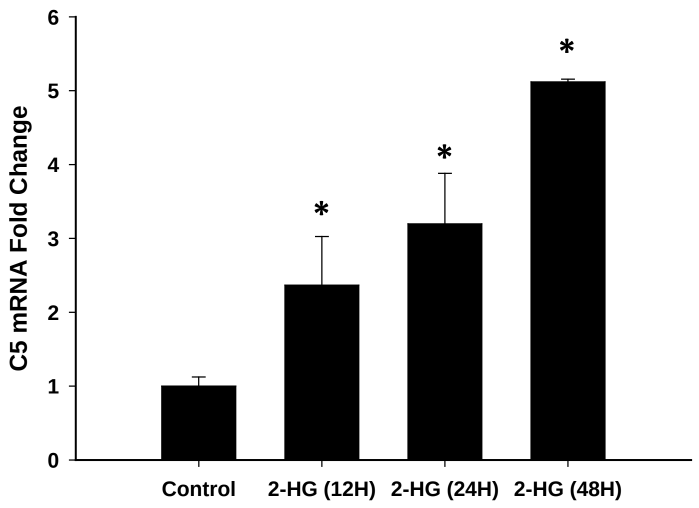

Supplementary Figure S4.

Octyl-*R*-2-HG increased complementary factor 5 (C5) expression in stromal cells.

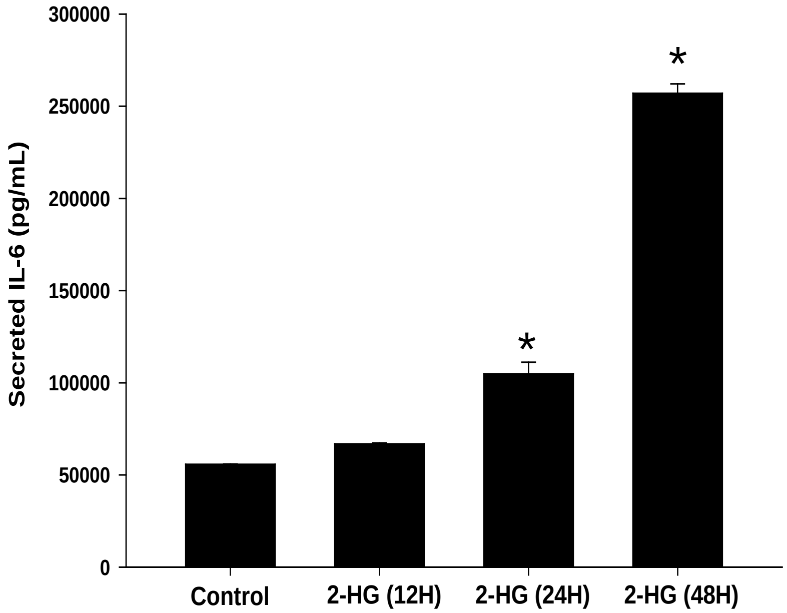

Supplementary Figure S5.

Octyl-*R*-2-HG increased secretion of IL-6 in the conditioned medium of stromal cells.

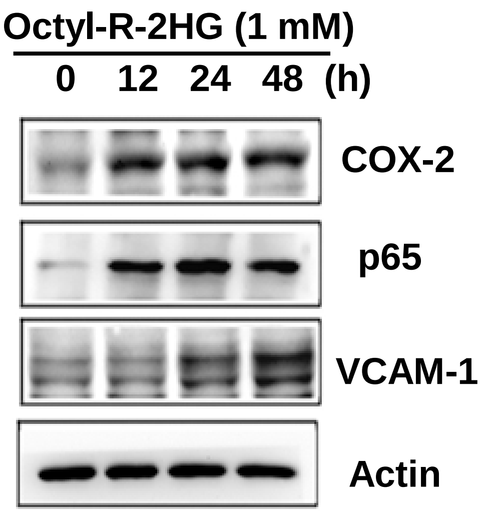

Supplementary Figure S6.

Octyl-*R*-2-HG increased the protein level of COX-2, p65 and VCAM-1 in stromal cells.

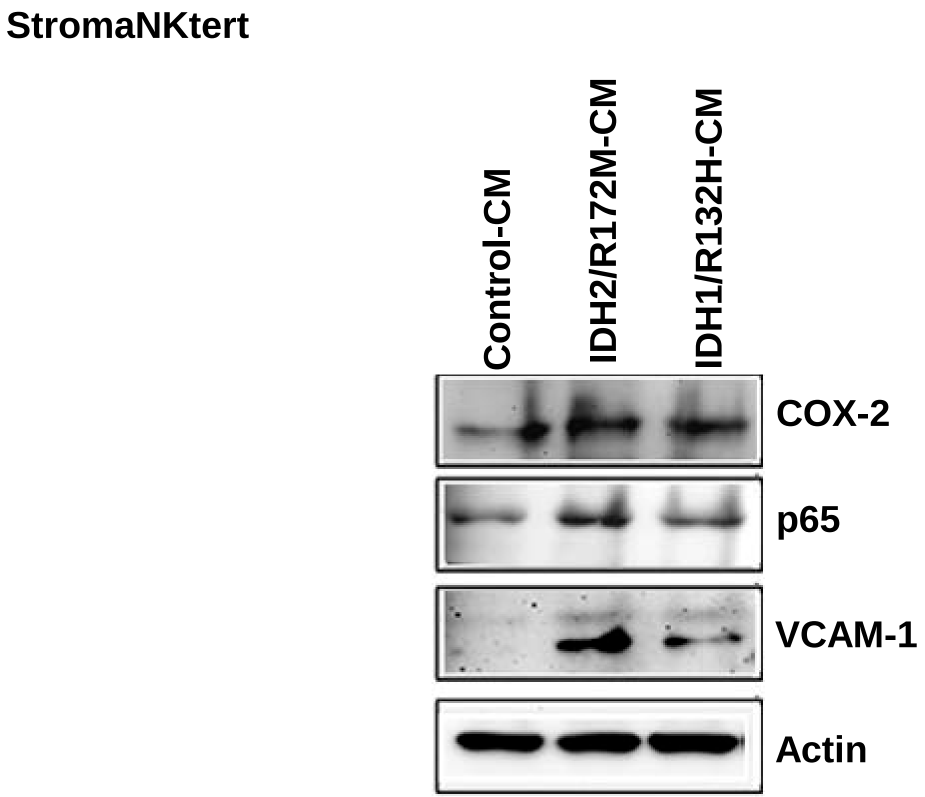

Supplementary Figure S7.

The conditioned media of KG-1a cells expressing different IDH mutants increased COX-2, p65 and VCAM-1 proteins in stromal cells. KG-1a cells were transfected with control or different IDH mutant expression vectors. After 72 h, the conditioned medium (CM) was collected to treat StromaNKtert cells and the protein level of COX-2, p65 and VCAM-1 was determined.

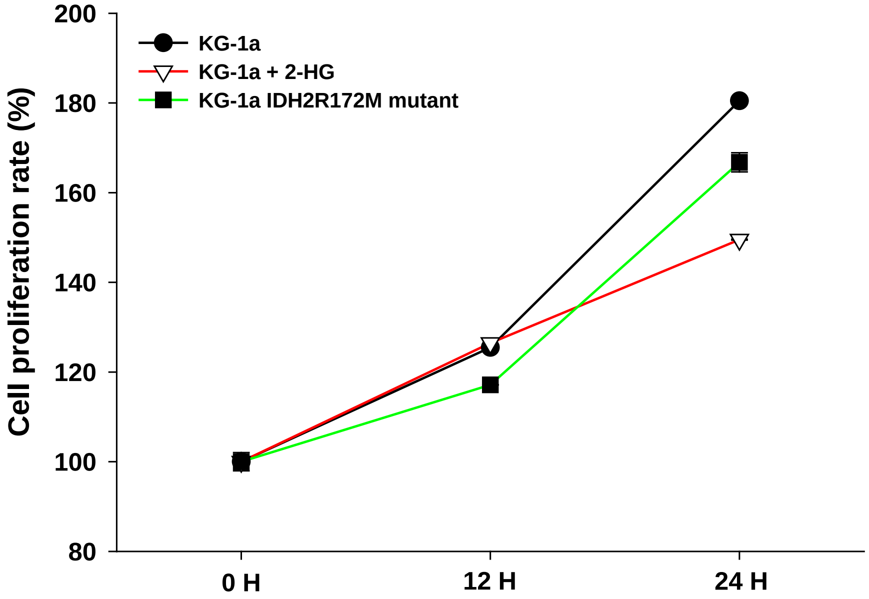

Supplementary Figure S8.

Treatment of *R*-2HG or expression of IDH2/R172 mutant did not increase the proliferation of KG-1a cells.

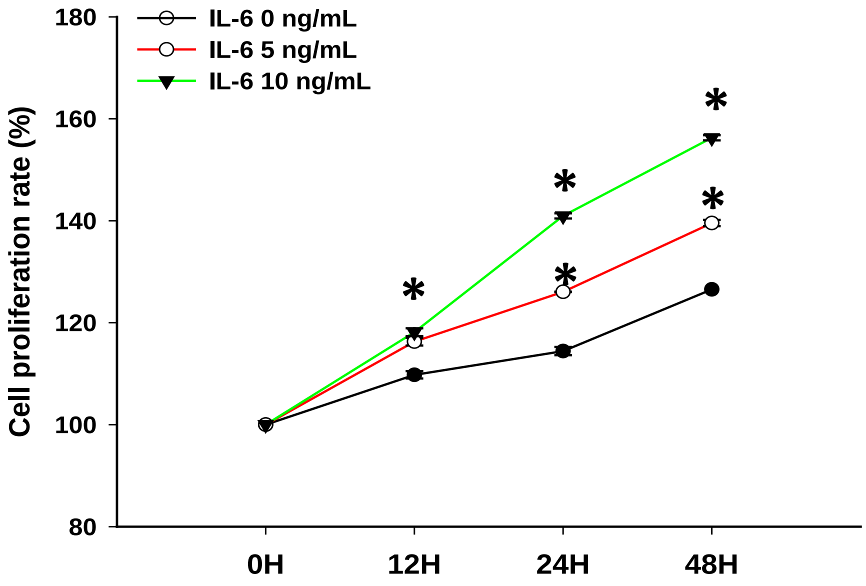

Supplementary Figure S9.

IL-6 stimulated the proliferation of KG-1a cells.

Supplementary Table S1: Information of three *IDH*-mutated AML patients

	Diagnosis[1]	Age/Gender	Cytogenetics	Treatment	Outcome
Patient 1	AML, M2	36/F	46 XX [25]	Chemotherapy, allogeneic HSCT[2]	Alive, disease free
Patient 2	AML, M1	37/F	46 XX [25]	Chemotherapy	Refractory, expired
Patient 3	AML, M1	50/F	47 XX, +11 [20]; 46 XX [2]	Chemotherapy	Relapsed, expired

[1] Based on FAB classification and [2] Hematopoietic stem cell transplantation

Supplementary Table S2: Antibodies and primer sequences used in this study

Antibody	Source	Source code
COX-2	Santa Cruz	SC-1745
p65	Invitrogen	510500
VCAM-1	GeneTex	GTX110684
Actin	Millipore	NAB1501
ERK	Cell Signaling	9102s
p-ERK	Cell Signaling	9101s
IκBα	Santa Cruz	SC-371
IKKα/β	Santa Cruz	SC-7607
PIN1	Santa Cruz	SC-46660
α-Tubulin	GeneTex	GTX628802
c-Myc	Santa Cruz	SC-764

Gene Name	Primer
COX-2	forward: 5’-GTCCTTTCAAGGAGAATGGTGC-3’
	reverse: 5’-GGTCTGGTGCCTGGTCTGATGATG-3’
IL-6	forward: 5’-CTTTCGTAACC GCACCCTGG-3’
	reverse: 5’-TCAGTGTCAGTGCGACTTCC-3’
VCAM-1	forward: 5’-CGTCTTGGTCAGCCCTTCCT-3’
	reverse: 5’- ACATTCATATACTCCCGCATCCTTC-3’
p65	forward: 5’-CACCTAGCTGCC AAAGAAGG-3’
	reverse: 5’-GGCACAACTCCTTCATCCTC-3’
SDF-1	forward: 5’-TCAGCCTGAGCTACAGATGC-3’
	reverse: 5’-CTTT AGCTTCGGGTCAATGC-3’
VLA4	forward: 5’-GAGTGCAATGCAGACCTTGA -3’
	reverse: 5’-TGGATTTGGCTCTGGAAAAC-3’
Actin	forward: 5’-TGTTACCAACTGGGACGACA-3’
	reverse: 5’-GGGGTGTTGAAGGTCTCAAA-3’
C5	forward: 5’-CCAAATGTGTGCGCCAGAAA-3’
	reverse: 5’-TGGGAACTCCTTTCGTCTGC-3’
